# Translation of nutrient recommendations into personalized optimal diets for Chinese urban lactating women by linear programming models

**DOI:** 10.1186/s12884-018-2008-6

**Published:** 2018-09-18

**Authors:** Kai Yu, Yong Xue, Wenzhi Zhao, Ai Zhao, Wenjun Li, Yumei Zhang, Peiyu Wang

**Affiliations:** 1 Department of Nutrition and Food Hygiene, School of Public Health, Peking University, Beijing, China; 2 CAS Key Laboratory of Pathogenic Microbiology and Immunology, Institute of Microbiology, Chinese Academy of Science, Beijing, China; 3 Department of Social Medicine and Health Education, School of Public Health, Peking University, Beijing, China; 4 Nestlé Nutrition Institute, Beijing, China

**Keywords:** Lactation mother, Linear programming, Nutrient recommendation, Personalized optimal diets

## Abstract

**Background:**

Lactating women need to consume a high-quality diet to replete nutrient stores depleted during pregnancy and to ensure sufficient nutrition for breastfeeding. However, several studies reported suboptimal dietary quality and nutrient intake of lactating mothers in China. The objectives of this study was to apply dietary modeling method to develop individualized optimal diets, which meet the nutrient requirements for lactating women in urban China.

**Methods:**

Data were collected from a sample of 576 lactating women from 0 to 240 days postpartum during the Maternal Infant Nutrition Growth study conducted between 2011 and 2012 in three cities including Beijing, Guangzhou, and Suzhou. Dietary intake data were collected with an interviewer-administered 24-h survey. Linear programming was applied to develop dietary plans that meet recommendations for lactation women in the China Dietary Reference Intakes 2013 and the Chinese Dietary Guideline 2016, while with least deviation from the observed dietary intake.

**Results:**

Through dietary modeling, individual optimal diets were developed for 576 lactating women. The optimal diets met all the food and nutrient intake constraints set in the linear programming models. The large difference between observed and optimized diets suggests that the nutrient needs of lactating mothers in China may only be met after substantial dietary changes. In addition, the analysis showed that it was difficult to meet the recommended intake for six nutrients: vitamin A, vitamin B1, vitamin B6, calcium, selenium, and dietary fiber. Moreover, four clusters in the optimized diets were identified by K-means cluster analysis. The four clusters confirmed that the optimal diets developed by linear programming could characterize the variety in dietary habits by geographical regions and duration of lactation.

**Conclusion:**

Linear programming could help translate nutrient recommendations into personal diet advices for a sample of urban lactating mothers from China. The study showed that dietary modeling is helpful to support healthy eating of lactation women by translating dietary guidelines into personalized meal plans.

**Trial registration:**

The Maternal Infant Nutrition Growth study was registered in ClinicalTrials.gov with identifier NCT01971671. Registration date October 29, 2013.

## Background

According to the recommendation on maternal nutrition from the International Federation of Gynecology and Obstetrics (FIGO), healthy eating during lactation period is essential to help mothers to rebuild body stores of nutrients depleted during pregnancy, as well as to conserve nutrient stores for ensuring supply of breast milk without compromising maternal nutrition reserve [1]. Considering the increasing nutritional need to support infant’s growth, the nutritive demands of lactation mothers are greater than non-lactating women [2]. However, previous studies from different countries reported suboptimal dietary quality and nutrient intake of lactation mothers [3–5].

The dietary pattern of a lactating mother is influenced by her cultural and social context. Reports from countries like Spain [6], UK [7], US [8], and Brazil [9] showed little or no change in dietary pattern from preconception to postpartum period. However, due to the influence of cultural beliefs such as postpartum “confinement period”, diet changes during pregnancy and postpartum periods were found in some ethnic groups [10]. In China, the confinement period is called *zuo yuezi* (literal meaning as “doing the month” or “sitting the month”), which includes a set of practices to guide diet, activity and hygiene based on traditional beliefs and theories [11]. Our previous study found that urban Chinese women within 5–30 day postpartum had a significant lower nutrient intake compared to those after 30 day postpartum [12]. The problem of inadequate nutrient intakes was also reported from a sample of lactating women within 90 day postpartum from Fujian Province in China [13]. Considering the multiple factors affecting dietary habits of lactating women, it is important to take into account cultural and individual preferences when providing dietary advices.

Dietary modeling is an approach to derive optimal diets by meeting an objective function subject to a set of constraints such as nutrient intake recommendation [14]. Based on linear programming, dietary modeling can help solve the complex problem of designing diets that meet nutrient recommendations while maintaining the local food habits and the intake of culture-specific foods [15]. The dietary modeling problem was first proposed by the Nobel Prize laureate George Stigler in 1945, when he tried to solve the cost minimization problem of designing a balanced diet plan that meets major nutrient recommendation [16]. Later, George Dantzig developed the theory of linear programming which formally solved this diet problem. With the availability of readily accessible computer technology, linear programming have been used for different purposes in nutrition and public health: translating nutrient recommendations into individual food choices [15, 17]; characterization of sustainable diets meeting a list of nutritional, economics and environmental constraints [18–20]; development of diet-based intervention for developing countries solely based on local food available [21, 22]; assessment of compatibility between food- and nutrient-based recommendations [23]. Linear programming was also applied to generate nutrition advice for individuals participating in a large scale, randomized controlled trial to compare effect on dietary behavior change between personalized nutrition advices versus conventional population-based recommendation [24].

Considering the importance of nutrition for lactating mothers and the suboptimal nutrient intake for this group in China, individualized dietary advice maybe helpful to provide realistic dietary guidance. However, to the best of our knowledge, no previous study have reported for developing individualized optimal diets based on linear programming in Chinese population. Therefore, the objective of the present study is to develop individual optimal dietary plan for a sample of urban Chinese lactating mothers. Based on linear programming, the aim of the optimal diets are to achieve nutrient recommendation in Dietary Reference Intakes (DRIs), while accounting for individual food intakes. In addition, by characterizing the “optimal diets” derived from dietary modelling, we also want to specify the modification needed for meeting DRIs with minimal deviation from current diets.

## Methods

### Subjects

The dietary data of lactating mothers were obtained from the Maternal Infant Nutrition and Growth Study (MING Study), which is a cross-sectional survey on dietary and nutrition status of pregnant women, lactating women, infants and toddlers aged 0–3 years. In the MING study, lactating women were selected from three cities (Beijing, Suzhou, and Guangzhou) with a purposive sampling approach. The three cities were chosen for representing north (Beijing), middle (Suzhou) and south (Guangzhou) of China. In each city, one hospital and one community-based maternal and child health care center were randomly selected from a computer-generated hospital list. At each site, mothers at lactation period 0–240 days were randomly chosen according to registration information provided by hospital. For lactating mother in the period of 0–4 day postpartum, recruitment was conducted at the hospital. For subjects at lactating stage of 5–240 days, requests of interview were conducted by phone. The inclusion criteria are: healthy women aged between 18 and 45 years, with healthy full term delivery, without cardiovascular and metabolic diseases, without using any hormone in past three months and without postpartum depression.

The final sample size of lactating women in MING study was 580. By excluding four subjects with daily energy intake lower than 200 kcal in the observed diets, 576 lactating mothers were included in the present study, including 214 from Beijing, 180 from Guangzhou, and 182 from Suzhou. The numbers of subjects from different lactation stages are 106 from 0 to 4 day postpartum, 187 from 5 to 30 day postpartum, and 283 from 31 to 240 day postpartum.

### Dietary data collection and preparation

Dietary intake status of lactating mothers was assessed by one cycle 24 h recall. During the interview, trained interviewers asked participants to report all food and beverages including seasonings and supplements consumed the day before interview. Quantity of item consumed and time or description of the meal were recorded. Measurement aids including standard bowls, plates and spoons, as well as a picture booklet of common foods consumed in China were used to help quantification of food items. All food, beverages and supplements were recorded. The energy and nutrient intake of lactating mothers were calculated based on the Chinese Food Composition Table [25].

In the present study, all food items recorded were categorized into 41 food groups according to the food group codes in the Chinese Food Composition Table. The resulted intakes in food groups are defined as “observed diets”. The nutrient profiles of the 41 food groups were established according to the method in previous studies [15, 26]. To develop nutrient profiles for the 41 food groups, relative consumption of each food item under corresponding food groups was estimated based on the 24 h recall data. We assigned a weight to the nutrients from each food item that corresponded to the percentage consumption in its food group. The nutrient profile for each food group was then established.

### Description of optimized model

To identify optimal diets that satisfy nutrition recommendation with least departure from current diet, linear programing approach is applied in this study.

Linear programming is a mathematical approach employed to identify the optimal solution of an objective function, which is dependent on a set of decision variables restricted by various linear constraints [27, 28]. In the current study, the objective function was to minimize differences in food intake between the observed and the optimized food group intakes by lactating mothers.

According to previous studies [15, 27], the objective function was mathematically described as the sum of the absolute values of differences between the intake of each food group in the optimized diets and that in the observed diets divided by that in the observed diets, as to standardize the difference across food groups:

$$ Y={\sum}_{i=1}^{i=41}\left|\frac{x_i^{opt}-{x}_i^{obs}}{x_i^{obs}}\right|, $$

where *Y* is the objective function to minimize, ${x}_i^{opt}$denotes the quantity (g) of food group *i* in the optimized diets, and ${x}_i^{obs}$ is the quantity (g) of food group *i* intake in the surveyed food data.

Because of its nature as an absolute value, *Y* was nonlinear. To meet the requirement of linear programming, *Y* was transformed into a linear function using the goal programming approach [21]. New decision variables ≥0 and representing the positive (*P*_1_ to *P*_41_) and negative (*N*_1_ to *N*_41_) deviation from the observed food group quantity were created and defined as follows:

$$ If\kern0.5em {x}_i^{opt}<\kern0.5em {x}_i^{obs}, then\kern0.5em Ni=\frac{x_i^{obs}-{x}_i^{opt}}{x_i^{obs}}\kern0.5em and\kern0.5em {P}_i=0 $$

$$ If\kern0.5em {x}_i^{opt}>\kern0.5em {x}_i^{obs}, then\kern0.5em Ni=0\kern0.24em and\kern0.5em Pi=\frac{x_i^{obs}-{x}_i^{opt}}{x_i^{obs}} $$

$$ If\kern0.5em {x}_i^{opt}=\kern0.5em {x}_i^{obs}, then\kern0.5em Ni=0\kern0.5em and\kern0.5em {P}_i=0 $$

$$ Subject\kern0.5em to:\kern0.5em Pi- Ni=\frac{x_i^{opt}-{x}_i^{obs}}{x_i^{obs}}\kern0.5em $$

The new linear function call *Y′* was expressed as the sum of deviational variables and was minimized:

$$ {Y}^{\hbox{'}}={\sum}_{i=1}^{i=41}{P}_i-{N}_i $$

Each food group in the objective function was linked to the nutrient profile of this food group established for the present study as mentioned above. The linkage of food group intake to nutrient profile database allowed the model to calculate and check at all times whether nutritional constraints were satisfied.

For every lactating mother included in this study, a model was developed to identify a new individually modeled diet that fulfills all the nutritional constraints.

### Nutritional constraints for linear programming models

Nutritional constraints were defined to allow linear programming models to seek optimal solutions. The sets of nutritional constraints include total dietary energy, nutritional targets, and maximal quantities of food groups.

The total dietary energy constraint was set equal to 2300 kcal/d, as recommended as the Estimated Energy Requirement (EER) for lactating mother in Chinese Dietary Reference Intakes (DRIs) 2013 [29]. The adoption of an isocaloric approach was due to lack of physical activity level data in the survey; thus, the recommendation of energy intake taking into account of physical activity level could not be considered.

Nutrient constraints were set for 21 nutrients with recommended intake values from Chinese DRIs 2013. The lower limits of specific nutrients followed the corresponding value of Recommended Nutrient Intake (RNI) and Adequate Intake (AI). The upper limits were in accordance with Tolerable Upper Intake Level (UL) or Proposed Intakes for Preventing Non-Communicable Chronic Diseases (PI-NCD). For carbohydrate and fat, the ranges defined as Acceptable Macronutrient Distribution Ranges (AMDR) were applied as lower and upper bounds. Table 1 shows the details of nutrient constraints for each of the 21 nutrients.

**Table 1:** Nutrient constraints included in the linear programming optimization model

Dietary Reference Intake Constraints	Lower limits based on RNI, AI or AMDR^a^	Upper limits based on AMDR, PI-NCD or UL^b^
Energy (kcal/d)	2300 (EER)	
Macronutrients (AMDR)		
Energy supply from carbohydrate (%)	≥50	≤65
Energy supply from fat (%)	≥20	≤30
Nutrients with RNI or AI		
Protein (g/d)	≥80	
Vitamin B_1_(mg/d)	≥1.5	
Vitamin B_2_(mg/d)	≥1.5	
Vitamin C(mg/d)	≥150	≤2000
Vitamin E(μg/d)	≥17(AI)	≤700
Vitamin A(μgRAE/d)	≥1300	≤3000
Vitamin B_6_(mg/d)	≥1.7	≤60
Niacin (mg/d)	≥15	≤35
Calcium (mg/d)	≥1000	≤2000
Phosphorus (md/d)	≥720	≤3500
Potassium (mg/d)	≥2400 (AI)	
Magnesium (mg/d)	≥330	
Iron (mg/d)	≥24	≤42
Zinc (mg/d)	≥12	≤40
Selenium (μg/d)	≥78	≤400
Copper (mg/d)	≥1.4	≤8
Manganese (mg/d)	≥4.8 (AI)	≤11
Sodium (mg/d)	≥1500	≤2000(PI-NCD)
Dietary fiber (g/d)	≥25(AI)	

^a^Values are RNI except for energy (EER), carbohydrate (AMDR), fat (AMDR), manganese (AI), potassium (AI), vitamin E (AI), and dietary fiber (AI). ^b^Values are UL except for energy (EER), carbohydrate (AMDR), fat (AMDR), and sodium (PI-NCD)

The food group intake recommendations from Dietary Guideline for Chinese 2016 [30] were taken as reference to set up constraints for maximal quantities of each food group (Table 2). Food groups without recommendation were mainly processed foods. By considering typical serving size, the upper limits of solid food and sugar-sweetened beverages were defined as 100 g and 250 g, respectively.

**Table 2:** Food group intake constraints included in the linear programming model

Food group	Upper limit (g)	Food group	Upper limit (g)	Food group	Upper limit (g)
Wheat bun	400	Red & yellow vegetable	500	Egg	50
Wheat noodle	400	Other vegetable	500	Liquid milk	500
Bread	400	Pickle	500	Yogurt	500
Rice	400	Apple and pear	350	Milk powder	500
Wheat flour product (fried)	200	Banana	350	Soup	250
Wheat flour product (non-fried)	400	Processed fruit	350	Sugar-sweetened beverage	250
Potato	100	Other fruit	350	Fast food	100
Tuber	100	Citrus fruit	350	Fried snack	100
Coarse grain	150	Pork	75	Non-fried snack	100
Various beans	150	Poultry	75	Cake and ice-cream	100
Soybean	35	Aquatic product^a^	75	Plant oil	30
Nut	35	Other livestock meat	75	Animal oil	30
Millet	400	Organ meat	20	Condiment	6
Leafy vegetable	500	Processed meat	50		

^a^Aquatic products refer to fish, shrimps, crabs, shellfish and other fishery products

### Statistical analysis

Linear programming models were developed for each individual of the 576 lactation women in this study. The output data of optimized diets include total diet weights and changes from observed diets.

For each of the 41 food groups, the quantity in their optimized diets vs. the corresponding observed diets, the percentage of increase or decrease for lactating women who consumed given food group, as well as changes of nutrient intakes between observed and optimized diets were calculated. The contribution of food groups to nutrients was also estimated from the optimized diets. For estimating sources of nutrients from food groups in the optimal diets, the weighted percentage contribution of each food group was calculated by adding the amount of a given nutrient provided by each food group for all participants and dividing by the total intake of that nutrient consumed by all participants from all foods and beverages in the optimal diets.

To evaluate whether the optimized diets derived from linear programming could reflect distinctive dietary habits by geographical locations and duration of lactation, K-means cluster analysis was employed to determine dietary clusters following the approach of Thorpe et al. [31]. The K-means cluster analysis needs to specify the number of clusters prior to analysis. A cluster contained < 10% number of participants in the total sample was considered not having adequate statistical power. The final clusters with each accounting for at least 10% subjects were examined for interpretability to confirm.

For characteristics of participants, mean value with standard errors were used for continuous variables, and counts and percentages for categorical values. Descriptive and difference analyses were performed with SPSS version 20.0 (SPSS Inc., USA). All of the reported *p* values were 2-tailed, and those < 0.05 were considered to be statistically significant. Solver add-in from Excel 2013 (Microsoft Inc., USA) was used for linear programming according to the method described by Briend et al. [16].

## Results

Total and changes of diet weights between observed and optimized diets.

Optimized diets satisfying all nutritional constraints were obtained for every lactating mother. The total diet weights of optimized diets and the deviation from observed diets were shown in Table 3. The mean variance between observed and optimized diets was 1325 g, which suggests that the reach of optimal diets required substantial changes in food group intakes.

**Table 3:** Distribution of total diet weights of the optimized diets and variation from the observed diets

	Mean	P5^a^	P25	Median	P75	P95
Total diet weight of optimized diets (g)	1638 ± 8.51	1346	1487	1620	1752	2014
Different Cities (g)						
Beijing	1641 ± 15.55^ab^	1290	1462	1622	1785	2035
Guangzhou	1680 ± 13.27^a^	1409	1568	1664	1767	2000
Suzhou	1592 ± 14.16^b^	1351	1435	1549	1724	1922
Different lactation stages						
0-4d	1521 ± 16.29^c^	1271	1390	1543	1646	1797
5-30d	1629 ± 13.57^b^	1344	1488	1625	1745	1986
31-240d	1687 ± 12.49^a^	1388	1523	1667	1840	2084
Variation between the observed and the optimized diets (g)	1325 ± 17.94	784	1034	1231	1539	2148
Different cities						
Beijing	1304 ± 29.89^b^	723	1026	1209	1537	2153
Guangzhou	1473 ± 36.18^a^	811	1138	1354	1721	2424
Suzhou	1206 ± 22.82^c^	828	977	1155	1335	1873
Different lactation stages						
0-4d	1345 ± 32.58	952	1122	1246	1462	2032
5-30d	1368 ± 31.82	867	1069	1290	1568	2347
31-240d	1288 ± 27.18	748	972	1184	1513	2153

^a^P stands for distribution [5th percentile (p5), first quartile (P25), median, third quartile (p75), and 95th percentile (p95)]

Significant differences in variation between the observed and the optimized diets were found among different cities. Compared to subjects from Beijing and Suzhou, lactating women from Guangzhou exhibited the significantly higher food intake changes required to achieve optimized diets.

The total diet weights were significantly different among different lactation stages. The mean value of total diet weights were highest for subjects from 31 to 240 day postpartum, with significant differences compared with those from 0 to 4 days and 5–30 days.

### Analysis of changes between observed and optimized diets

Noticeable dietary changes were found between the observed and the optimized diets. Table 4 shows the comparison of food group consumption rates. The food group of organ meat is the only one with 100% consumption rate in the optimized diets, and organ meat was added in their optimized diets by 91% of participants. Three food groups with consumption rate reached 99% in the optimized diets, including leafy vegetable, condiment, and egg. Increased intakes of leafy vegetable in optimized diets were found in 97% of subjects, while condiment were reported to have 88% of subjects with decreased intakes. Pork (98%), red and yellow vegetable (97%), nut (97%), milk powder (96%), aquatic product (95%), coarse grain (90%) were the six food groups with consumption rate as 90% or above. Compared with observed diets, only six food groups had higher percentage of decreased intakes in optimized diets, including condiment, rice, plant oil, poultry, soup, and soybean. For food groups that were added in the optimized diets but absent in the observed diets, there were nine food groups with more than 50% subject reported an addition: milk powder (94%), organ meat (91%), nut (82%), coarse grain (80%), banana (72%), red and yellow vegetable (69%), various beans (66%), and wheat flour product (non-fried) (53%).

**Table 4:** Food group intake and consumption rates in the optimized diets and changes of consumption rates from the observed diets

Food group	Intake in optimized diets (g)	Consumption rate in optimized diets %	Consumption rate in observed diets %	Lactating mother with increased intake of this food group %	Lactating mother with decreased intake of this food group %	Lactating mother with food group added in optimized diets %
Organ meat	20	100	9	92	7	91
Leafy vegetable	320	99	53	97	2	46
Condiment	3	99	92	11	88	8
Egg	46	99	56	50	42	43
Pork	58	98	63	61	35	34
Red & yellow vegetable	180	97	28	95	2	69
Nut	28	97	14	90	7	82
Milk powder	28	96	1	94	1	94
Aquatic product	44	95	30	73	22	65
Coarse grain	97	90	10	88	3	80
Rice	137	88	83	26	58	5
Banana	64	84	12	75	9	72
Other vegetable	96	77	69	62	15	8
Wheat flour product (non-fried)	50	76	23	67	9	53
Various beans	34	75	10	73	2	66
Potato	35	64	15	57	6	50
Plant oil	4	63	88	6	84	2
Processed fruit	9	55	5	52	3	49
Wheat bun	22	54	23	39	15	31
Poultry	22	50	35	22	27	15
Non-fried snack	4	50	16	40	11	36
Bread	5	47	12	39	10	37
Wheat noodle	27	45	22	30	15	23
Fast food	11	43	17	32	9	27
Wheat flour product (fried)	1	42	4	39	3	39
Soup	75	40	35	12	26	5
Cake and ice-cream	3	37	5	35	3	33
Processed meat	8	37	7	32	4	30
Citrus fruit	38	36	15	32	3	21
Other livestock meat	5	33	8	27	7	26
Millet	28	31	22	18	13	9
Apple and pear	43	29	20	21	8	9
Fried snack	1	29	1	28	1	27
Soybean	8	27	25	8	19	2
Animal oil	0	26	3	24	3	24
Liquid milk	44	23	16	14	9	7
Tuber	8	22	13	14	7	9
Other fruits	20	19	13	14	5	7
Pickle	1	10	6	7	3	4
Sugar sweetened beverage	12	10	6	5	4	4
Yogurt	3	8	2	6	1	6

**Fig. 1:**
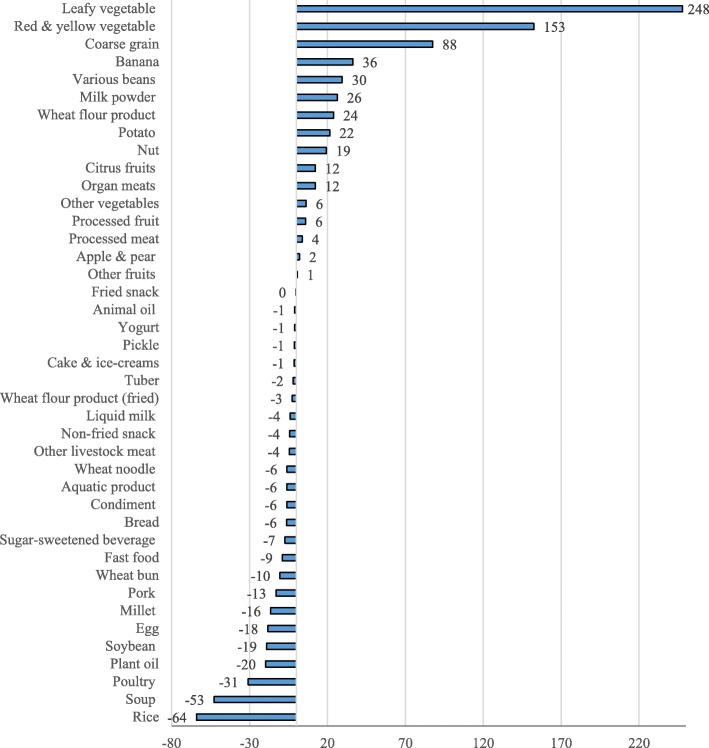
Change of food group intake between the observed and the optimized diets

Figure 1 presents the changes of food group intake quantities in the optimized diets from the observed diets. Among the 41 food groups, to achieve optimal diets that meet all nutrient recommendation in DRIs, increased intakes were needed for 16 food groups, and reduced quantities were found for 24 groups. Leafy vegetable showed the highest amount of increase in the optimized diet, followed by red and yellow vegetable, coarse grain, banana, various beans, milk powder, wheat flour product (non-fried), potato, nut, organ meat, as the top ten food groups with increased intakes compared to observed diets. For food groups with decrease in the optimized diets, the averaged amount of rice found the highest reduction as 64 g. Other food groups with average reduced intakes higher than 15 g in optimal diets include soup, poultry, plant oil, soybean, egg, and millet. The food group of fried snack was the only group without change between observed and optimized diets.

A comparison of energy and nutrient contents between the observed and optimized diets was shown in Table 5. The energy intakes in the optimized diets of the 576 lactating women all reached 2300 kcal/d as the energy intake constraint set in the linear program model. The contributions to energy by both carbohydrate (58.74%) and fat (27.45%) in the optimal diets met the recommended ranges in AMDR. While in the observed diets, the contribution to energy from both fat and carbohydrate were outside the recommended value in AMDR. In the optimal diets of all the 576 participants, all nutrients reached RNI or AI values. It should be noted that nutrient contents of six nutrients including vitamin A, vitamin B1, vitamin B2, vitamin B6, calcium, selenium, and dietary fiber just reached 100% of the DRIs value, suggesting these six nutrients as limiting nutrients in the optimized diets.

**Table 5:** Comparison of nutrient contents between the observed and optimized diets

DRIs		Mean	
		Observed diets	Optimized diets
Energy (kcal/d)	2300	1836	2300
Macronutrients(AMDR)			
Carbohydrate(%)	50–60	49.56	58.74
Fat (%)	20–30	36.35	27.45
Nutrients with RNI or AI^a^			
Protein (g/d)	80	74.11	104.33
VitaminB_1_(mg/d)	1.50	0.96	1.51
VitaminB_2_(mg/d)	1.50	1.07	2.17
Vitamin C(mg/d)	150	86.20	251.67
Vitamin E(μg/d)	17	23.73	26.82
Vitamin A (μgRAE/d)	1300	520.02	1300.14
VitaminB_6_(mg/d)	1.7	0.87	1.74
Niacin(mg/d)	15	17.13	27.63
Ca(mg/d)	1000	466.72	1004.47
P(md/d)	720	1014.12	1652.28
K(mg/d)	2400	1749.71	3808.69
Mg(mg/d)	330	272.85	595.44
Fe(mg/d)	24	21.07	32.47
Zn(mg/d)	12	10.88	15.81
Se(μg/d)	78	57.31	78.47
Cu(mg/d)	1.4	1.85	3.09
Mn(mg/d)	4.8	4.83	8.11
Na(mg/d)	1500	4114.29	1970.26
Fiber (g/d)	25	9.74	25.39

^a^Nutrients based on RNI include protein, calcium, phosphorus, magnesium, iron, selenium, zinc, vitamin A, vitamin B_1_, vitamin B_2_, vitamin B_6_, niacin, vitamin C. Nutrients based on AI include potassium, sodium, manganese, vitamin E and dietary fiber

### Sources of nutrients and dietary patterns of optimized diets

To understand how linear programming model modifies diets and generates optimized diets, the present study analyzed sources of nutrients and dietary clusters in the optimized diets.

Table 6 shows the sources of energy and nutrients in the optimized diets. Considering the recommendation to have at least 12 different kinds of foods on a daily basis from Chinese Dietary Guideline 2016 [30], we presented the contribution of energy and nutrients from top 12 food groups as well as top five food groups.

**Table 6:** Contribution to energy and nutrients by food groups in the optimized diets

	Contribution %						
	Top 12	Top 5	No.1	No. 2	No. 3	No. 4	No. 5
Energy	69.63	41.71	Coarse grain	Pork	Rice	Nut	Liquid milk
			13.01	8.32	7.08	6.72	6.58
Protein	74.14	43.84	Aquatic product	Pork	Coarse grain	Liquid milk	Egg
			11.29	9.03	8.76	7.98	6.78
Dietary fiber	92.53	62.08	Coarse grain	Other vegetable	Leafy vegetable	Nut	Various beans
			22.35	12.21	10.24	9.07	8.21
Vitamin B_1_	78.55	48.21	Coarse grain	Pork	Wheat flour product (non-fried)	Red & yellow vegetable	Nut
			17.53	12.00	6.95	6.11	5.60
Vitamin B_2_	78.41	51.37	Liquid milk	Leafy vegetable	Organ meat	Milk powder	Egg
			17.84	9.71	8.75	8.35	6.73
Niacin	77.41	46.75	Nut	Coarse grain	Pork	Aquatic product	Other vegetable
			13.78	11.36	7.92	7.49	6.21
Vitamin C	90.89	74.99	Leafy vegetable	Red & yellow vegetable	Other vegetable	Other fruit	Citrus fruit
			28.64	17.95	15.09	8.09	5.58
Vitamin E	81.80	53.48	Nut	Various beans	Coarse grain	Apple & pear	Other vegetable
			19.77	9.38	9.14	7.90	7.29
Ca	88.28	68.44	Liquid milk	Leafy vegetable	Other vegetables	Milk powder	Aquatic product
			28.73	18.46	8.04	7.49	5.72
P	77.66	46.41	Coarse grain	Liquid milk	Aquatic product	Nut	Leafy vegetable
			14.22	12.47	8.22	6.20	5.30
K	79.34	47.67	Red & yellow vegetable	Leafy	Liquid milk	Coarse grain	Banana
			12.35	11.22	8.80	8.23	7.07
Mg	79.58	49.64	Coarse grain	Nut	Leafy vegetable	Banana	Other vegetable
			15.86	10.44	9.90	7.60	5.85
Fe	73.27	46.16	Leafy vegetable	Other vegetable	Coarse grain	Various beans	Organ meat
			11.83	10.96	9.94	7.24	6.19
Zn	73.39	40.42	Coarse grain	Pork	Liquid milk	Leafy vegetable	Aquatic product
			11.91	7.59	7.36	6.95	6.62
Se	79.87	55.03	Aquatic product	Organ meat	Egg	Pork	Liquid milk
			17.78	13.14	10.06	7.27	6.77
Cu	72.93	40.16	Nut	Coarse grain	Various beans	Other vegetable	Leafy vegetable
			10.69	8.94	8.83	5.93	5.77
Mn	82.25	50.80	Coarse grain	Nut	Rice	Banana	Other vegetable
			13.59	12.17	8.52	8.43	8.09
Vitamin A(RAE)	97.87	88.95	Organ meat	Red & yellow vegetable	Leafy vegetable	Egg	Liquid milk
			30.34	27.35	16.70	9.57	5.00
Vitamin B_6_	83.71	52.50	Red & yellow vegetable	Banana	Leafy vegetable	Nut	Coarse grain
			16.35	12.11	10.87	7.25	5.92
Fat	85.92	66.55	Pork	Nut	Liquid milk	Egg	Coarse grain
			23.61	18.44	12.72	6.80	5.19
Na	91.66	72.37	Condiment	Leafy vegetable	Liquid milk	Egg	Aquatic product
			50.68	6.44	5.31	5.05	4.88
Carbohydrate	78.64	51.16	Coarse grain	Rice	Wheat flour product (non-fried)	Banana	Wheat bun
			18.71	10.80	8.11	6.85	6.69

It can be seen from Table 6 that the Top 12 food group accounted for 69.63% of energy in the optimized diets. For nutrients, the contribution of nutrients from the corresponding top 12 food groups ranged from 72.93 to 97.87%. For top five food groups, the ranges of contribution to energy and nutrients were between 40.16 and 88.95%. Among individual food group, condiment showed the highest percentage with its contribution to sodium as 50.68%. Food groups with contribution to certain nutrients higher than 20% also include: organ meat for vitamin A (30.34%), liquid milk for calcium (28.73%), leafy vegetable to vitamin C (28.64%), red and yellow vegetable for vitamin A (27.35%), pork to fat (23.61%), and coarse grain to fiber (22.35%).

From Table 6, we can also calculate the frequency of different food groups appearing in the top five contributors for energy and nutrients in the optimized diets, as below: coarse grain (16 times), leafy vegetable (13 times), liquid milk (11 times), nut (11 times), other vegetable (9 times), pork (7 times), aquatic product (7 times), egg (6 times), red and yellow vegetable (5 times), banana (5 times), organ meat (4 times), various beans (4 times), rice (3 times). There are 22 food group without presence in the top five contributors of energy or any nutrient in the optimized diets, indicating that these foods may have limited role to nutrient supply in the optimal diets.

Four reasonably sized clusters (> 10% of sample size) were identified from the optimized diets (Table 7). Cluster 1(*n* = 66) was characterized by higher intake of wheat bun, millet, apple and pear, and liquid milk, with least intake of milk powder. Lactating women within cluster 2(*n* = 160) was characterized by red and yellow vegetable and milk powder, as well as lowest intake of bread and soup. The cluster with highest number of lactation mothers was Cluster 3 (*n* = 209), which showed higher intakes of wheat noodle, leafy vegetable, poultry, and banana, as well as lowest intake of other vegetables. Cluster 4 (*n* = 141) was characterized by higher intake of rice, citrus fruit, while with lowest intakes of millet and banana.

**Table 7:** Mean of food group intakes in the optimized diets by dietary clusters^a^

Food groups	Cluster 1^b^	Cluster 2	Cluster 3	Cluster 4
	*N* = 66 (11.46%)	*N* = 160 (27.78%)	*N* = 209 (36.28%)	*N* = 141 (24.48%)
Wheat bun	**44.96** ^**a**^	28.50^b^	15.16^c^	13.02^c^
Wheat noodle	16.69^b^	19.31^b^	**38.79** ^**a**^	21.62^b^
Bread	8.88^a^	0.82^c^	6.04^ab^	4.61^b^
Rice	103.21^bc^	90.75^c^	131.39^b^	**211.89** ^**a**^
Wheat flour product (fried)	2.01	1.85	0.94	1.44
Wheat flour product (non-fried)	37.36^bc^	63.25^a^	55.82^ab^	34.23^c^
Potato	33.08	37.80	35.02	33.90
Tuber	9.39^ab^	6.16^b^	4.29^b^	16.64^a^
Coarse grain	96.88	92.89	98.45	97.94
Various beans	29.47	39.24	32.69	30.28
Soybean	10.19^a^	7.16^ab^	5.80^b^	10.08^a^
Nut	22.17^b^	28.55^a^	28.58^a^	27.39^a^
Millet	**55.32** ^**a**^	29.40^b^	31.62^b^	7.40^c^
Leafy vegetable	177.88^c^	165.62^c^	**465.03** ^**a**^	347.83^b^
Red & yellow vegetable	266.85^b^	**315.03** ^**a**^	79.05^d^	134.90^c^
Other vegetables	154.65^a^	127.02^ab^	42.62^c^	112.82^b^
Pickle	0.45	1.57	0.77	1.05
Apple & pear	**82.82** ^**a**^	38.85^b^	31.07^b^	47.42^b^
Banana	62.09^b^	61.66^b^	**82.85** ^**a**^	39.3^c^
Citrus fruit	37.64^b^	17.07^bc^	8.88^c^	**104.14** ^**a**^
Other fruits	32.94^a^	31.39^a^	7.03^b^	20.73^ab^
Processed fruit	10.57^ab^	12.35^a^	6.69^b^	6.08^b^
Pork	52.97^b^	54.30^b^	57.05^b^	**64.67** ^**a**^
Poultry	15.49^b^	14.74^b^	28.54^a^	22.94^ab^
Aquatic product	39.25^b^	48.96^a^	35.56^b^	52.58^a^
Other livestock meat	8.98^a^	2.44^b^	6.41^a^	3.24^b^
Organ meat	19.95	19.66	19.92	19.56
Processed meat	8.19	5.90	9.69	7.10
Egg	46.87^ab^	47.40^a^	47.11^a^	43.07^b^
Liquid milk	**276.57** ^**a**^	9.22^b^	13.16^b^	18.64^b^
Yogurt	0.00	5.21	0.50	4.42
Milk powder	9.92^c^	**48.38** ^**a**^	22.15^b^	23.87^b^
Cake & ice-cream	6.11	2.56	3.45	1.14
Non-fried snack	1.65	2.93	5.32	3.78
Fast food	14.99	8.40	13.17	10.58
Fried snack	0.63	0.86	0.57	0.41
Soup	85.05^a^	45.22^b^	85.77^a^	87.95^a^
Sugar-sweetened beverage	19.35	8.36	8.24	17.10
Plant oil	3.07	2.80	4.62	3.61
Animal oil	0.17	0.19	0.38	0.19
Condiment	2.84	2.83	2.63	2.75

^a^Mean values between cluster without common letter differ, *P*<0.05 tested with ANOVA and bonferroni post hoc. The cluster with significantly higher intake for corresponding food group are marked bold. ^b^Characteristic food groups for different clusters: Cluster 1-wheat bun, millet, apple and pear, and liquid milk; cluster 2-red and yellow vegetables and milk powder; cluster 3-wheat noodle, leafy vegetables, poultry, banana; cluster 4-rice, citrus fruits

The distribution of lactating mothers in different clusters as shown in Table 8. The results showed that the optimized diets for lactating mothers from Beijing in the northern China tend to be in Cluster 1 and Cluster 2, while mothers from the south in both Guangzhou and Suzhou are more likely be in Cluster 3 and Cluster 4. Table 8 also presents distribution of subjects from different lactating stages in different clusters. For lactating mothers at 0–4 day postpartum, 48.11% of the optimized diets were in Cluster 3, and 36.79% were in Cluster 2. Cluster 3 (48.66%) also accounts for the highest percentage in lactation stage 5–30 day postpartum, followed by Cluster 2 (22.99%). But in 31–240 day postpartum, 36.04% of the optimized diets for lactating women were classified as Cluster 4, higher than the other three clusters. In addition, subjects classified as Cluster 1 increased from 6.60% in 0–4 day postpartum to 12.30% and 12.72% in 5–30 days and 31–240 days.

**Table 8:** Percentage of lactation mothers classified to dietary clusters

		Cluster 1^a^	Cluster 2	Cluster 3	Cluster 4
Difference cities					
Beijing	N	46	89	53	26
*N* = 214	%	21.50	41.59	24.77	12.15
Guangzhou	N	10	36	85	49
*N* = 180	%	5.56	20.00	47.22	27.22
Suzhou	N	10	35	71	66
*N* = 182	%	5.49	19.23	39.01	36.26
Different lactation stages					
0-4d	N	7	39	51	9
*N* = 106	%	6.60	36.79	48.11	8.49
5-30d	N	23	43	91	30
*N* = 187	%	12.30	22.99	48.66	16.04
31-240d	N	36	78	67	102
*N* = 283	%	12.72	27.56	23.67	36.04

^a^Characteristic food groups for different clusters: Cluster 1-wheat bun, millet, apple and pear, and liquid milk; cluster 2-red and yellow vegetables and milk powder; cluster 3-wheat noodle, leafy vegetables, poultry, banana; cluster 4-rice, citrus fruits

## Discussion

Adequate maternal nutrition in lactating stage is critical to ensure optimal growth of infants through breastmilk as well as maintaining nutrient storage in lactating mothers, therefore nutrient needs of lactating women are higher than non-lactating women [30]. However, the specific nutrition needs during lactation may remain as a challenge because of various reasons like cultural practice of confinement diets [1], lack of awareness and poor knowledge [32], in addition to inertia to dietary guidance identified in general population due to an array of cultural, socio-economic, behavioral factors [33]. Diet modeling approach such as linear programming offers the possibility to translate nutrient recommendation into realistic and personalized food advices [17]. Optimized diets generated by linear programming models in the present study could provide recommended micronutrients and macronutrients for sampled individual lactating mothers in the correct amounts and proportion, which meets the definition of an adequate diet by FIGO [1]. To our best knowledge, this study is the first one to employ linear programming to translate nutrient-based recommendation for Chinese lactating women into personalized diets that satisfy nutrient recommendation.

Our results show that substantial dietary modification from current diets of Chinese urban lactating women is needed to fulfill the nutritional recommendation for lactation stage. The average value of food group changes between the observed and the optimized diets in the current study was 1325 g. In a study on designing optimal food intake patterns to achieve nutritional goals for Japanese adults by linear programing, the average amount of dietary changes from the observed to the optimized diets were 1262 g/d for female aged 30-49y, and 1028 g/d for male aged 30-49y [15]. In another study based on a representative sample of French population, individual diet modeling was applied to translate nutrient recommendations into weekly food plan [17]. The median increase in total diet weight was 1332 g/week, with the 5th percentile as 302 g/week and the 95th percentile as 1893 g week. The same group also reported that for the vast majority of French adults, it was mathematically impossible to design an optimized diet meeting all nutrient recommendation without expanding the diversity of foods consumed on a weekly basis [34]. Similar changes were identified to develop an optimal diet for the individuals of Chinese urban lactating women in our study.

The approach of reaching an optimal diets by linear programming model is to replace energy-dense food groups by nutrient-dense food groups. The 16 food groups with increased intakes in the optimized diets were nutrient-dense, while the 24 food groups with decreased amounts tend to be energy-dense. As the most nutrient-dense food [23], leafy vegetable and red-yellow vegetable showed highest increases in quantity. The increased amount of coarse grains in the optimized diets could be justified by the fact that coarse grains served as the top 5 contributors for energy and 15 nutrients. The replacement of refined grains like rice, millet, and wheat flour product by coarse grain with higher nutrient density in the current study is consistent with the results by Okubo et al. [15]. The consumption rates of food groups in the optimized diets showed that some specific food groups are key to achieving nutrient adequacy. There are 10 food groups with consumption rates reaching 90% or above. With the exception of condiment, the other nine food groups showed increased of intakes or addition of food groups compared to the observed diets. These nine food groups covered the major food categories recommended in the Chinese Dietary Pagoda [30], and may serve as core food groups in healthy dietary patterns for lactating women.

Food groups that could supply limiting nutrients may be prioritized by linear programming to ensure a solution for meeting all constraints. For example, the consumption rate of banana reached 84%, with 72% as new addition in the optimized diets. The reason to have increased amount of banana intake may be due to its contribution to vitamin B6 (12.11%), which was identified as a limiting nutrient. Banana is also the top 5 contributor for magnesium, potassium, manganese, and energy. The increase of food groups that satisfy limiting nutrients in the optimization process could also be seen from organ meat. The consumption rate of organ meats is 100% in the optimized diets, as the only food group that is recommended for every lactating mothers. Indeed, the intake of organ meat reached the upper limit of food group intake constraint set as 20 g. Organ meat is the major supplier of three limiting nutrients including vitamin A (30.34%), selenium (13.14%), and vitamin B2 (8.75%), as well as top 5 food group sources for iron.

The K-means cluster analysis identified four clusters in the optimized diets, confirming that nutrient recommendations can be achieved in different ways, depending on the initial individual food patterns [17]. The optimized diets of lactating women showed distinctive differences between Beijing in the northern China and cities of Suzhou and Guangzhou from the south part of China. A national representative dietary survey also reported the geographical difference of dietary patterns between southern and northern China [35].

The objective function to minimize departure from observed diets ensured the optimized diets to reflect the differences in food choices at different lactation stages. Cluster 3 is featured with higher intakes of wheat noodle, leafy vegetable, poultry, and banana. Cluster 3 also accounts for almost half of the subjects in the first month postpartum, followed by a decline to 24% after the first month. While the number of lactating women with an optimized diets classified as Cluster 4 increased from 8% in 0–4 day postpartum to 16% in 5–30 day postpartum and 36% in 31–240 day postpartum. Therefore, Cluster 3 maybe serve as the confinement diets, which also agreed with cultural practices in the southern part of China to have wheat noodle and poultry especially chicken at the first month after giving birth [11]. A longitudinal study on Chinese dietary pattern from 1991 to 2009 with a sample of 9253 Chinese adults identified two stable dietary patterns including a traditional southern dietary pattern, which was characterized by high intakes of rice, fresh leafy vegetables, low-fat red meat, pork, organ meats, poultry and fish/seafood and low intakes of wheat flour and maize/coarse grains [36]. The traditional southern dietary pattern is similar to Cluster 4 in the optimized diets in terms of both characteristic food groups as well as features of subjects. Therefore, by setting an objective function as minimized deviation from current diets, the optimized diets could be nutritionally adequate but still reflects the food choices by geographical region and duration of lactation.

The major strength of this study was to apply linear programming for the first time in a Chinese lactation population to develop individualized, optimal diets that meet nutrient recommendations with least departure from the current diet. The result showed that suggests that the nutrient needs of lactating mothers in China may only be met after substantial dietary changes and with the addition of nutrient-dense food. As shown among European adults in the Food4Me European randomized controlled trial, personalized nutrition advices enabled by linear programming approaches produced larger and more appropriate changes in dietary behavior than non-personalized, conventional healthy eating guidelines [24]. So this study provides knowledge foundation to implement digital nutrition approach for large scale intervention to provide individualized recommendation to support healthier eating behavior of lactating women in China.

Several limitations of this study warrant mention. First, the observed diets used in the current study was based on one-day 24 h recall, which limited the ability to generate optimized diet plans for longer period due to the absence of information about day-to-day variation. In addition, due to limitation of Chinese Food Composition Table, the current study did not estimate some nutrients relevant to maternal and infant nutrition such as fatty acids, folic acids, vitamin D. Moreover, the optimized diets were built on the hypothesis that the observed diets may reflect individual preference. But in reality, many factor could contribute to the choices of food, so information about actual personal preference will clearly help improve the approach. Lastly, this study does not include intervention component. An intervention study may be needed to show improvement in dietary quality through personalized nutrition recommendation enabled by linear programming approach, compared to conventional, non-personalized dietary guideline.

## Conclusions

In conclusion, linear programming could help translate nutrient recommendations into personal diet advices for a sample of urban lactating mothers from China. Substantial changes including increasing diversity and intakes of nutrient-dense foods were needed to achieve optimal diets. Such information can provide references for establishing dietary guidelines and meal plans for lactation mothers, and also support for maintaining a healthy diet with adequate nutrient supply for both breastfeeding and mothers’ own nutrition storage.

## Abbreviations

AI: Adequate Intake
AMDR: Acceptable Macronutrient Distribution Ranges
DRIs: Dietary Reference Intakes
EER: Estimated Energy Requirement
FIGO: International Federation of Gynecology and Obstetrics
MING Study: Maternal Infant Nutrition and Growth Study
PI-NCD: Proposed Intakes for Preventing Non-Communicable Chronic Diseases
RNI: Recommended Nutrient Intake
UL: Tolerable Upper Intake Level

## References

[1] Hanson Mark A., Bardsley Anne, De-Regil Luz Maria, Moore Sophie E., Oken Emily, Poston Lucilla, Ma Ronald C., McAuliffe Fionnuala M., Maleta Ken, Purandare Chittaranjan N., Yajnik Chittaranjan S., Rushwan Hamid, Morris Jessica L. (2015). The International Federation of Gynecology and Obstetrics (FIGO) recommendations on adolescent, preconception, and maternal nutrition: “Think Nutrition First”#. International Journal of Gynecology & Obstetrics.

[2] Picciano Mary Frances (2003). Pregnancy and Lactation: Physiological Adjustments, Nutritional Requirements and the Role of Dietary Supplements. The Journal of Nutrition.

[3] Giammarioli Stefania, Sanzini Elisabetta, Ambruzzi Amalia Maria, Chiarotti Flavia, Fasano Gemma (2002). Nutrient Intake of Italian Women during Lactation. International Journal for Vitamin and Nutrition Research.

[4] Fowles Eileen R., Walker Lorraine O. (2006). Correlates of Dietary Quality and Weight Retention in Postpartum Women. Journal of Community Health Nursing.

[5] Moran L J, Sui Z, Cramp C S, Dodd J M (2012). A decrease in diet quality occurs during pregnancy in overweight and obese women which is maintained post-partum. International Journal of Obesity.

[6] Cucó G, Fernández-Ballart J, Sala J, Viladrich C, Iranzo R, Vila J, Arija V (2005). Dietary patterns and associated lifestyles in preconception, pregnancy and postpartum. European Journal of Clinical Nutrition.

[7] Crozier Sarah R., Robinson Siân M., Godfrey Keith M., Cooper Cyrus, Inskip Hazel M. (2009). Women's Dietary Patterns Change Little from Before to During Pregnancy. The Journal of Nutrition.

[8] Sotres-Alvarez Daniela, Herring Amy H., Siega-Riz Anna-Maria (2013). Latent Transition Models to Study Women's Changing of Dietary Patterns From Pregnancy to 1 Year Postpartum. American Journal of Epidemiology.

[9] Dos Santos Q, Sichieri R, Marchioni DM, Verly Junior E. Brazilian pregnant and lactating women do not change their food intake to meet nutritional goals. BMC Pregnancy Childbirth. 2014;14:186. doi: 10.1186/1471-2393-14-186. PMID: 24890188.[CrossRef PubMed]. 10.1186/1471-2393-14-186 PMC4049461 24890188

[10] Chen Ling-Wei, Low Yen Ling, Fok Doris, Han Wee Meng, Chong Yap Seng, Gluckman Peter, Godfrey Keith, Kwek Kenneth, Saw Seang-Mei, Soh Shu E, Tan Kok Hian, Chong Mary Foong Fong, van Dam Rob M (2013). Dietary changes during pregnancy and the postpartum period in Singaporean Chinese, Malay and Indian women: the GUSTO birth cohort study. Public Health Nutrition.

[11] Raven JH, Chen Q, Tolhurst RJ, Garner P. Traditional beliefs and practices in the postpartum period in Fujian Province, China: a qualitative study. BMC Pregnancy Childbirth. 2007; 7: 8. doi: 10.1186/1471-2393-7-8. PMID: 17584930. [CrossRef PubMed]. 10.1186/1471-2393-7-8 PMC1913060 17584930

[12] Zhao Ai, Xue Yong, Zhang Yumei, Li Wenjun, Yu Kai, Wang Peiyu (2016). Nutrition Concerns of Insufficient and Excessive Intake of Dietary Minerals in Lactating Women: A Cross-Sectional Survey in Three Cities of China. PLOS ONE.

[13] Chen H, Wang P, Han Y, Ma J, Troy FA, Wang B. Evaluation of dietary intake of lactating women in China and its potential impact on the health of mothers and infants. BMC Women’s Health. 2012;12:18. doi:10.1186/1472-6874-12-18. PMID: 22800437. [CrossRef PubMed]. 10.1186/1472-6874-12-18 PMC3419073 22800437

[14] Irz Xavier, Leroy Pascal, Réquillart Vincent, Soler Louis-Georges (2016). Beyond Wishful Thinking: Integrating Consumer Preferences in the Assessment of Dietary Recommendations. PLOS ONE.

[15] Okubo H, Sasaki S, Murakami K, Yokoyama T, Hirota N, Notsu A, et al. Designing optimal food intake patterns to achieve nutritional goals for Japanese adults through the use of linear programming optimization models. Nutr J. 2015; 14:57. doi: 10.1186/s12937-015-0047-7. PMID: 26048405. [CrossRef PubMed]. 10.1186/s12937-015-0047-7 PMC4470056 26048405

[16] Briend André, Darmon Nicole, Ferguson Elaine, Erhardt Juergen G. (2003). Linear Programming: A Mathematical Tool for Analyzing and Optimizing Children's Diets During the Complementary Feeding Period. Journal of Pediatric Gastroenterology and Nutrition.

[17] Maillot Matthieu, Vieux Florent, Amiot Marie Josèphe, Darmon Nicole (2009). Individual diet modeling translates nutrient recommendations into realistic and individual-specific food choices. The American Journal of Clinical Nutrition.

[18] Wilson Nick, Nghiem Nhung, Ni Mhurchu Cliona, Eyles Helen, Baker Michael G., Blakely Tony (2013). Foods and Dietary Patterns That Are Healthy, Low-Cost, and Environmentally Sustainable: A Case Study of Optimization Modeling for New Zealand. PLoS ONE.

[19] Donati M, Menozzi D, Zighetti C, Rosi A, Zinetti A, Scazzina F. Towards a sustainable diet combining economic, environmental and nutritional objectives. Appetite. 2016, 106: 48–57. doi: 10.1016/%20j.appet.2016.%2002.151. PMID: 26921487. [CrossRef PubMed]. 10.1016/j.appet.2016.02.151 26921487

[20] Horgan GW, Perrin A, Whybrow S, Macdiarmid JI. Achieving dietary recommendations and reducing greenhouse gas emissions: modelling diets to minimise the change from current intakes. Int J Behav Nutr Phys Act. 2016, 13:46. doi: 10.1186/s12966-016-0370-1. PMID: 27056829. [CrossRef PubMed]. 10.1186/s12966-016-0370-1 PMC4823893 27056829

[21] Fahmida Umi, Santika Otte, Kolopaking Risatianti, Ferguson Elaine (2014). Complementary Feeding Recommendations Based on Locally Available Foods in Indonesia. Food and Nutrition Bulletin.

[22] Hlaing Lwin Mar, Fahmida Umi, Htet Min Kyaw, Utomo Budi, Firmansyah Agus, Ferguson Elaine L. (2015). Local food-based complementary feeding recommendations developed by the linear programming approach to improve the intake of problem nutrients among 12–23-month-old Myanmar children. British Journal of Nutrition.

[23] Maillot Matthieu, Drewnowski Adam (2012). A Conflict Between Nutritionally Adequate Diets and Meeting the 2010 Dietary Guidelines for Sodium. American Journal of Preventive Medicine.

[24] Celis-Morales C, Livingstone KM, Marsaux CF, Macready AL, Fallaize R, O'Donovan CB, et al. Effect of personalized nutrition on health-related behaviour change: evidence from the Food4me European randomized controlled trial. Int J Epidemiol. 2016. doi: 10.1093/ije/dyw186 PMID: 27524815. [CrossRef PubMed]. 10.1093/ije/dyw186 27524815

[25] Yang Y (2009). China food composition second edition.

[26] Marcoe Kristin, Juan WenYen, Yamini Sedigheh, Carlson Andrea, Britten Patricia (2006). Development of Food Group Composites and Nutrient Profiles for the MyPyramid Food Guidance System. Journal of Nutrition Education and Behavior.

[27] Masset Gabriel, Monsivais Pablo, Maillot Matthieu, Darmon Nicole, Drewnowski Adam (2009). Diet Optimization Methods Can Help Translate Dietary Guidelines into a Cancer Prevention Food Plan. The Journal of Nutrition.

[28] Anderson A. M., Earle M. D. (1983). Diet Planning in the Third World by Linear and Goal Programming. The Journal of the Operational Research Society.

[29] China Nutrition Society (2014). China DRIs handbook (2013).

[30] China Nutrition Society (2016). Dietary guideline for Chinese (2016).

[31] Thorpe MG, Milte CM, Crawford D, McNaughton SA. A comparison of the dietary patterns derived by principal component analysis and cluster analysis in older Australians. Int J Behav Nutr Phys Act. 2016;13:30. doi:10.1186/s12966-016-0353-2. PMID: 26928406. [CrossRef PubMed]. 10.1186/s12966-016-0353-2 PMC4772350 26928406

[32] Charlton Karen, Yeatman Heather, Lucas Catherine, Axford Samantha, Gemming Luke, Houweling Fiona, Goodfellow Alison, Ma Gary (2012). Poor Knowledge and Practices Related to Iodine Nutrition during Pregnancy and Lactation in Australian Women: Pre- and Post-Iodine Fortification. Nutrients.

[33] Webb D, Byrd-Bredbenner C. Overcoming consumer inertia to dietary guidance. Adv Nutr 2015 6(4):391–396. doi: 10.3945/an.115.008441 PMID: 26178023. [CrossRef PubMed]. 10.3945/an.115.008441 PMC4496743 26178023

[34] Maillot Matthieu, Vieux Florent, Ferguson Elaine F., Volatier Jean-Luc, Amiot Marie Josèphe, Darmon Nicole (2009). To Meet Nutrient Recommendations, Most French Adults Need to Expand Their Habitual Food Repertoire. The Journal of Nutrition.

[35] He Y., Ma G., Zhai F., Li Y., Hu Y., Feskens E. J.M., Yang X. (2009). Dietary Patterns and Glucose Tolerance Abnormalities in Chinese Adults. Diabetes Care.

[36] Batis Carolina, Sotres-Alvarez Daniela, Gordon-Larsen Penny, Mendez Michelle A., Adair Linda, Popkin Barry (2013). Longitudinal analysis of dietary patterns in Chinese adults from 1991 to 2009. British Journal of Nutrition.

